# Clinical value of MRI in evaluating and diagnosing of humeral lateral condyle fracture in children

**DOI:** 10.1186/s13018-021-02894-5

**Published:** 2022-01-06

**Authors:** Andreas Rehm, Joshua C. Y. Ong, Elizabeth Ashby

**Affiliations:** Paediatric Division, Department of Paediatric Orthopaedics, Cambridge University Hospitals NHS Trust, Hills Road, Cambridge, CB2 0QQ UK

**Keywords:** Lateral humeral condyle fracture, Lateral condyle humerus fracture, Humeral lateral condyle fracture, Elbow arthrogram, Elbow arthrography, Ultrasound, Paediatric elbow trauma, Pediatric elbow trauma, Lateral condyle fracture

We read with interest the recent publication by Qi and colleagues [1]. Qi et al. [1] stated that frontal and lateral radiographs of the elbow are still the first choice when diagnosing humeral lateral condyle fractures (HLCF). In contrary to the former, Bland et al. [2] reported that the internal oblique radiograph (IOR) is the most reliable and useful view to measure HLCF displacement and Edmonds et al. [3] identified that it is the most predictive view of subsequent displacement, compared to the antero-posterior (APR), lateral (LR) and external oblique view. Edmonds et al. [3] reported that 13 of 140 fractures with < 2 mm displacement at diagnosis displaced within a mean 7-day follow-up, requiring delayed surgery, which resulted in good clinical outcomes and low complication rates, no different to those who had early surgery.

Li et al. [4, 5] described the use of transverse ultrasound (US) to assess articular cartilage integrity in HLCFs as a simple technique without sedation, which can be performed independently by an orthopaedic surgeon and reported that the optimum cutoff to differentiate between stable and unstable fractures might be greater than 2 mm. The authors [4] assessed 39 HLCFs with 2–4 mm displacement on APRs and LRs, 14 without cartilage disruption were treated non-operatively without further displacement, and 16 with cartilage disruption had surgery. Nine others with cartilage disruption were treated non-operatively, but 4 of these showed further displacement during follow-up. The latter study indicates that fractures with 2–4 mm displacement with intact articular cartilage can be treated safely non-operatively.

Qi et al. [1] did not mention if the MRI scans were done under general anaesthetic or sedation. Xu et al. [6] reported that even when a multimedia supportive approach (“MRI-am-a-hero”) was used, the sedation rate for MRI scans of children aged 4–7 years was 47.5% and without this approach 64.3%.

Qi et al. [1] stated that US, arthrogram and arthroscopy are invasive and cannot be used routinely to evaluate HLCFs because of high cost, the need for sedation or poor reproducibility of results, which is contradicted by Li et al. [4, 5]. US machines are widely available within A&E and theatre departments nowadays, which would potentially allow for US to be used in the management of HLCFs as described by Li et al. [4, 5] at no extra cost and for the decision about operative or non-operative management to be made already in A&E. MRI in contrary is expensive, requires sedation in a large number of 4–7-year-old children and delays diagnosis and surgery. Qi et al. [1] reported a mean > 24 h delay to obtain MRI scans after radiographs were taken, which means that children might have to be admitted to hospital, blocking valuable bed space or families having to spend extra time and effort to travel to the hospital for the MRI.

Qi et al. [1] reported that they measured displacement with an accuracy of 1/100th of a millimetre, with interobserver intraclass correlation coefficients (ICC) for radiographs and MRIs between 0.87 and 0.92 with a significant mean difference of 0.17 mm for LRs and 0.15 mm for APRs, respectively.

Bland et al. [2] recorded 2.4% interobserver ICC disagreement for IORs (APR: 8.2%; LR: 6.1%) if disagreement was defined as > 2 mm difference between measurements, which increased to 15.1% (APR: 22.5%; LR: 17.4%) if a threshold of > 1 mm was used with a standard error of the mean (SEM) of ~1 mm, indicating that a further reduction of the level of difference to 0.5, 0.1 or even 0.01 mm would make it unreliable to measure the amount of displacement down to the difference. Qi et al. [1] stated they had, especially with the latter authors not having used IORs.

In conclusion, US would be a cheaper and more readily available imaging modality to assess HLCFs than MRI, possibly allowing early treatment decision making in A&E. Considering that Qi et al. [1] have not used IORs but less reliable APRs and LRs, it is possible that there might not be a difference between MRI and radiograph false positive/negative rates and measurements of displacement. Li et al.’s [4, 5] findings indicate that the threshold between non-operative and operative treatments could possibly be moved from the 2 mm mark into the 2–4 mm range, with the potential to treat more patients non-operatively, reducing risks and improving outcomes for patients [7], with reduced costs for already stretched health services.

## Data Availability

Not applicable.

## Abbreviations

A&E: Accident and emergency
APR: Anteroposterior radiograph
HLCF: Humeral lateral condyle fractures
ICC: Intraclass correlation coefficient
IOR: Internal oblique radiograph
LR: Lateral radiograph
MRI: Magnetic resonance imaging
SEM: Standard error of the mean
US: Ultrasound
